# Periodontal disease and effects of antipsychotic medications in patients newly diagnosed with schizophrenia: a population-based retrospective cohort

**DOI:** 10.1017/S204579601900043X

**Published:** 2019-09-17

**Authors:** Kai-Fang Hu, Pei-Shan Ho, Yu-Hsiang Chou, Jui-Hsiu Tsai, Chung-Hung Richard Lin, Hung-Yi Chuang

**Affiliations:** 1 Department of Dentistry, Kaohsiung Municipal Ta-Tung Hospital, Kaohsiung Medical University, Kaohsiung, Taiwan; 2 Division of Periodontics, Department of Dentistry, Kaohsiung Medical University Hospital, Kaohsiung Medical University, Kaohsiung, Taiwan; 3 Division of Medical Statistics and Bioinformatics, Department of Medical Research, Kaohsiung Medical University Hospital, Kaohsiung Medical University, Kaohsiung, Taiwan; 4 Department of Oral Hygiene, College of Dental Medicine, Kaohsiung Medical University, Kaohsiung, Taiwan; 5 Department of Psychiatry, Dalin Tzu Chi Hospital, Buddhist Tzu Chi Medical Foundation, Chia-Yi, Taiwan; 6 Ph.D. Program in Environmental and Occupation Medicine, (Taiwan) National Health Research Institutes and Kaohsiung Medical University, Kaohsiung, Taiwan; 7 Department of Computer Science and Engineering, National Sun Yat-sen University, Kaohsiung, Taiwan; 8 Department of Public Health, Kaohsiung Medical University, Kaohsiung, Taiwan; 9 Department of Occupational Medicine, Kaohsiung Medical University Hospital, Kaohsiung Medical University, Kaohsiung, Taiwan

**Keywords:** Antipsychotics, early prevention, hyposalivation, periodontal disease, schizophrenia

## Abstract

**Aim:**

Compared with the general population, individuals with schizophrenia have a higher risk of periodontal disease, which can potentially reduce their life expectancy. However, evidence for the early development of periodontal disease in schizophrenia is scant. The current study investigated risk factors for periodontal disease in patients newly diagnosed with schizophrenia.

**Methods:**

We identified a population-based cohort of patients in Taiwan with newly diagnosed schizophrenia who developed periodontal disease within 1 year of their schizophrenia diagnosis. Treatment with antipsychotics and other medications was categorised according to medication type and duration, and the association between medication use and the treated periodontal disease was assessed through logistic regression.

**Results:**

Among 3610 patients with newly diagnosed schizophrenia, 2373 (65.7%) had an incidence of treated periodontal disease during the 1-year follow-up. Female sex (adjusted odds ratios [OR] 1.40; 95% confidence interval [CI] 1.20–1.63); young age (adjusted OR 0.99; 95% CI 0.98–0.99); a 2-year history of periodontal disease (adjusted OR 2.45; 95% CI 1.84–3.26); high income level (adjusted OR 2.24; 95% CI 1.64–3.06) and exposure to first-generation (adjusted OR 1.89; 95% CI 1.54–2.32) and secondary-generation (adjusted OR 1.33; 95% CI 1.11–1.58) antipsychotics, anticholinergics (adjusted OR 1.24; 95% CI 1.03–1.50) and antihypertensives (adjusted OR 1.91; 95% CI 1.64–2.23) were independent risk factors for periodontal disease. Hyposalivation – an adverse effect of first-generation antipsychotics (FGAs) (adjusted OR 2.00; 95% CI 1.63–2.45), anticholinergics (adjusted OR 1.27; 95% CI 1.05–1.53) and antihypertensives (adjusted OR 1.90; 95% CI 1.63–2.22) – was associated with increased risk of periodontal disease. Therefore, hypersalivation due to FGA use (adjusted OR 0.72; 95% CI 0.59–0.88) was considered a protective factor.

**Conclusions:**

The current study highlights that early prevention of periodontal disease in individuals with schizophrenia is crucial. Along with paying more attention to the development of periodontal disease, assessing oral health regularly, helping with oral hygiene, and lowering consumption of sugary drinks and tobacco, emphasis should also be given by physicians to reduce the prescription of antipsychotics to the extent possible under efficacious pharmacotherapy for schizophrenia.

## Introduction

Mortality risks associated with physical illnesses, especially cardiometabolic health, in patients with schizophrenia are attracting increased attention (Mitchell *et al*., 2013; Carney *et al*., 2016). By contrast, the periodontal health of patients with schizophrenia has remained overlooked (Arnaiz *et al*., 2011; Wey *et al*., 2016). Compared with the general population, patients with schizophrenia have a high incidence of periodontal disease (Kenkre and Spadigam, 2000; Ramon *et al*., 2003; Hu *et al*., 2016). Although periodontal disease is not an acutely life-threatening disease, an increasing body of evidence supports its association with cardiovascular disease, stroke, metabolic syndrome, pulmonary disease and adverse pregnancy outcomes (Pihlstrom *et al*., 2005; Cullinan and Seymour, 2013).

Periodontal disease – including gingivitis and periodontitis – is inflammation of the periodontium due to a persistent bacterial infection that leads to the breakdown of connective tissue and bone – a major cause of tooth loss in adults (Pihlstrom *et al*., 2005; Thomson *et al*., 2012; Ji *et al*., 2015). In the general population, recognised risk factors for periodontal disease include aged, male sex, race, unhealthy lifestyle (poor oral hygiene, smoking and alcohol use), poor nutrition (inadequate dietary consumption of calcium and vitamin D), systemic diseases (obesity, metabolic syndrome, osteoporosis, diabetes mellitus and HIV/AIDS), psychosocial stress, genetic factors and medications (Albandar, 2002; Pihlstrom *et al*., 2005; Thomson *et al*., 2012; Genco and Borgnakke, 2013). Antipsychotics and other medications affect salivary secretion often causing hyposalivation or hypersalivation. Salivary secretion dysfunction aggravates periodontal disease (Sekine *et al*., 1999; Hashimoto *et al*., 2012; Eltas *et al*., 2013). Only a few studies have assessed the link between antipsychotics and periodontal disease in patients with schizophrenia to date (Gopalakrishnapillai *et al*., 2012; Eltas *et al*., 2013). Therefore, the current study investigated this link.

Most research on periodontal disease and schizophrenia has been restricted to cross-sectional designs and small-to-medium samples; they have also focused on chronic schizophrenia (Arnaiz *et al*., 2011; Gurbuz *et al*., 2011; Teng *et al*., 2011; Gopalakrishnapillai *et al*., 2012; Eltas *et al*., 2013; Shetty and Bose, 2014; Nayak *et al*., 2016; Wey *et al*., 2016). The literature cannot fully explain the progress of development of periodontal disease in individuals with schizophrenia, especially the early stages of development. Given the lack of extensive literature, we identified a population-based cohort of patients newly diagnosed with schizophrenia from the Taiwan National Health Insurance Research Database (NHIRD), determined how many developed periodontal diseases within 1 year of their diagnosis, and identified risk factors associated with periodontal disease.

## Methods

### Study source and participants

Detailed descriptions of the Taiwan NHIRD sample and study procedures have previously been published (Hu *et al*., 2016; Lin *et al*., 2018). In summary, we employed the 1995–2010 NHIRD data, a subset composed of 1 million randomly sampled beneficiaries drawn in 2000. The Internal Review Board approved the study and informed consent was waived because we used de-identified medical information from the NHIRD.

We performed a cohort study of patients who were newly diagnosed with schizophrenia between 1 January 2000 and 31 December 2009. The patients were diagnosed based on the International Classification of Disease, Ninth Revision, Clinical Modification (ICD-9-CM) code 295 by at least two psychiatrists and such patients had been treated with antipsychotics for more than 3 months. The index date was defined as the date of the schizophrenia diagnosis. Exclusion criteria were as follows: HIV/AIDS, diabetes mellitus, chronic pulmonary disease, osteoporosis and alcoholism before the index date (because of the potential confounding factors for periodontal disease) (Albandar, 2002; Pihlstrom *et al*., 2005; Hu *et al*., 2016). Additionally, a 2-year history of periodontal disease before the index date was recorded.

### Incidence of treated periodontal disease after their index date

Claim records of periodontal disease of the participants comprised both the periodontal disease-related diagnoses (ICD-9-CM codes 523.0–523.5, 523.8 and 523.9) and anatomical therapeutic chemical codes diagnosed by dentists (91001C, 91003C, 91004C, 91006C–91008C, 91009B, 91010B, 91011C–91013C, 91104C, 92033C, P4001C and P4002C). The incidences of periodontal disease in participants diagnosed with and treated for periodontal disease within 1 year after their index date were recorded. We followed up all participants for 1 year until a diagnosis of periodontal disease, end of follow-up in medical records, death or the end of 2010.

### Exposure to antipsychotics and other medications

Appendix Table A1 presents antipsychotics and other medications in this study. We acquired these medication data from prescription files and estimated the pharmacotherapy duration based on the dosing regimen of each participant and the number of units dispensed. All medications were classified into four categories: first-generation antipsychotics (FGAs), second-generation antipsychotics (SGAs), anticholinergics and antihypertensives. Exposure to antipsychotics and other medications was recorded if a participant was prescribed the same medication for at least 4 weeks during the 1-year follow-up. Co-medications were considered as concomitant drugs that were simultaneously prescribed with antipsychotics and other medications.

The risk of periodontal disease for each antipsychotic and other medication was evaluated to identify adverse effects (hyposalivation and hypersalivation) following usage. The adverse effects of continuous medication use on periodontal disease were assessed. The potential adverse effects of antipsychotics and other medications are listed in Appendix Table A2 (Friedlander and Marder, 2002; Scully and Bagan, 2004; Muench and Hamer, 2012; Vinayak *et al*., 2013).

### Statistical analysis

All statistical analyses were performed using SAS statistical software (v 9.1, SAS Institute, Cary, NC, USA). Patients with schizophrenia who did and did not develop periodontal disease were analysed using the Pearson's *χ*^2^ test after stratification by sex, age, geographical region, income level, medical prescriptions and a 2-year history of periodontal disease. To analyse the independent effect of schizophrenia on the risk of developing periodontal disease, we used logistic regression after adjustment for sex, age, geographical region, income level, concomitant medical prescriptions, 2-year history of periodontal disease, potentially associated risk factors and index date. Moreover, we performed hierarchical logistic modelling using SAS GLMMIX to mitigate potential collinearity among sexes, geographical region and income level (Dai *et al*., 2006). We used logistic regressions for evaluating the individual odds ratios (ORs) of periodontal disease related to the adverse effects of antipsychotics and other medications and potentially associated risk factors. A two-sided statistical significance level of *p* < 0.05 was used in all analyses.

## Results

During the study period, 3610 patients were newly diagnosed with schizophrenia. The mean age at presentation was 34.7 years (standard deviation [s.d.] 14.3). Overall, 3295 (91.3%) patients belonged to a low income level class, and 2058 (57.0%) were prescribed multiple antipsychotics. Of all the enrolled patients, 367 (10.2%) had a 2-year history of periodontal disease before their respective index date (Table 1). During the 1-year follow-up period, 2373 (65.7%) patients received periodontal disease treatment: 300 had a 2-year history of periodontal disease and 2073 exhibited no such history (Table 2).

**Table 1.** Baseline characteristics of patients newly diagnosed with schizophrenia between 2000 and 2009 (*n* = 3610)

Baseline characteristics	Values
Age, mean (s.d.)	34.7 (14.3)
Sex, *N* (%)	
Male	1893 (52.4)
Female	1717 (47.6)
Monthly income (NT$), *N* (%)	
No income (0)	1082 (30.0)
Low income (<28 000)	2213 (61.3)
High income (≧28 000)	315 (8.7)
Geographical region of Taiwan, *N* (%)	
Northern	1788 (49.5)
Central	693 (19.2)
Southern	1031 (28.6)
Eastern and others	98 (2.7)
A 2-year history of periodontal disease, *N* (%)	367 (10.2)
Prescriptions, *N* (%)	
FGAs^a^	3003 (83.2)
SGAs^a^	2665 (73.8)
Anticholinergics	2809 (77.8)
Antihypertensives	2250 (62.3)

s.d., standard deviation; NT$, New Taiwan dollar.

a Including antipsychotic polypharmacy.

**Table 2.** Occurrence of treated periodontal disease among patients with newly diagnosed schizophrenia during 1-year follow-up

	Occurrence of periodontal disease	No. of occurrences of periodontal disease
With a 2-year past history of periodontal disease, *N* (%)	300 (81.7)	67 (18.3)
Without a 2-year past history of periodontal disease, *N* (%)	2073 (63.9)	1170 (36.1)

Table 3 presents the adjusted ORs associated with the demographics, clinical characteristics and prescriptions for the risk of treated periodontal disease in all patients, as determined using logistic regression. Female sex (adjusted OR 1.40; 95% confidence interval [CI] 1.20–1.63; *p* < 0.001), younger age (adjusted OR 1.02; *p* < 0.001), 2-year history of periodontal disease (adjusted OR 2.45; 95% CI 1.84–3.26; *p* < 0.001) and high income level (adjusted OR 2.24; 95% CI 1.64–3.06; *p* < 0.001) were independent risk factors for periodontal disease. Compared with nonusers, the ORs of FGA, SGA, anticholinergic and antihypertensive users are 1.89 (95% CI 1.54–2.32; *p* < 0.001), 1.33 (95% CI 1.14–1.58; *p* = 0.001), 1.24 (95% CI 1.03–1.50; *p* = 0.025) and 1.91 (95% CI 1.64–2.23; *p* < 0.001), respectively, after adjustment for sex, age, 2-year history of periodontal disease, income level, geographical region and the index date.

**Table 3.** Risk of periodontal disease in patients with newly diagnosed schizophrenia during 1-year follow-up

	OR^a^	95% CI	*p*
Age	0.99	0.98–0.99	<0.001
Sex (female *v*. male)	1.40	1.20–1.63	<0.001
Monthly income			
Low income (*v*. no income)	0.98	0.82–1.16	0.783
High income (*v*. no income)	2.24	1.64–3.06	<0.001
Geographical region of Taiwan			
Central (*v*. Northern)	1.03	0.84–1.25	0.802
Southern (*v*. Northern)	0.71	0.60–0.84	<0.001
Eastern and others (*v*. Northern)	0.68	0.44–1.06	0.088
A 2-year history of			
Periodontal disease (*v*. no)	2.45	1.84–3.26	<0.001
Prescriptions			
FGAs (*v*. no)	1.89	1.54–2.32	<0.001
SGAs (*v*. no)	1.33	1.11–1.58	0.001
Anticholinergics (*v*. no)	1.24	1.03–1.50	0.025
Antihypertensives (*v*. no)	1.91	1.64–2.23	<0.001

OR, odds ratio; CI, confidence interval.

a After adjustment for age, sex, income level, geographical region, 2-year history of periodontal disease, index date and concomitant prescriptions.

Furthermore, we analysed the adjusted OR for treated periodontal disease following potential hyposalivation or hypersalivation attributable to antipsychotic or other medication use (Table 4). FGA-induced hyposalivation was associated with an increased risk of treated periodontal disease (adjusted OR 2.00; 95% CI 1.63–2.46; *p* < 0.001). Similarly, anticholinergic- and antihypertensive-induced hyposalivation were associated with risks of treated periodontal disease (adjusted OR 1.27; 95% CI 1.05–1.53; *p* = 0.015 *v*. adjusted OR 1.90; 95% CI 1.63–2.22; *p* < 0.001, respectively). Hence, FGA-induced hypersalivation in patients with treated periodontal disease was identified as a protective factor (adjusted OR 0.72; 95% CI 0.59–0.88; *p* = 0.001).

**Table 4.** Adjusted ORs of periodontal disease in patients with newly diagnosed schizophrenia due to potential hyposalivation and hypersalivation caused by antipsychotics and other medications

Prescriptions	OR^a^	95% CI	*p*
FGAs			
Hyposalivation	2.00	1.63–2.46	<0.001
Hypersalivation	0.72	0.59–0.88	0.001
SGAs			
Hyposalivation	1.24	0.96–1.60	0.104
Hypersalivation	1.10	0.87–1.38	0.423
Anticholinergics			
Hyposalivation	1.27	1.05–1.53	0.015
Antihypertensives			
Hyposalivation	1.90	1.68–2.22	<0.001

OR, odds ratio; CI, confidence interval.

a After adjustment for age, sex, income level, geographical region, 2-year history of periodontal disease, index date and adverse effects of concomitant prescriptions.

## Discussion

According to a review of the relevant literature, this is the first study assessing the early development of periodontal disease in patients with schizophrenia. Data of 3610 patients newly diagnosed with schizophrenia between 2000 and 2009 were analysed. During the 1-year follow-up period, approximately two-thirds of these patients underwent periodontal disease treatment. Young age; female sex; high-income level; 2-year history of periodontal disease and exposure to FGAs, SGAs, anticholinergics and antihypertensives were independent risk factors for periodontal disease in the patients newly diagnosed with schizophrenia. Moreover, FGA-, anticholinergic- and antihypertensive-induced hyposalivation were associated with an increased risk of periodontal disease. Hence, hypersalivation caused by FGAs was considered a protective factor.

### Incidence of periodontal disease in schizophrenia

The incidence of periodontal disease in patients with schizophrenia varies with ethnicity, time, socioeconomic status, psychophysical condition, definition and length of illness and assessment tools (Angelillo *et al*., 1995; Tang *et al*., 2004; Pihlstrom *et al*., 2005; Arnaiz *et al*., 2011; Gurbuz *et al*., 2011; Teng *et al*., 2011). The following three studies – including the current study – among ethnic Chinese populations are considered as examples (Tang *et al*., 2004; Teng *et al*., 2011). A cross-sectional survey of oral health in central Taiwan using the community periodontal index (CPI) showed that 90% of psychiatric inpatients (schizophrenia: 61%, length of illness: approximately 6 years) had poor periodontal health (Teng *et al*., 2011). A dental study in Hong Kong demonstrated that 98.5% of inpatients with chronic schizophrenia (length of illness: 20 years) had poor periodontal health, as assessed using the standardised dental evaluation of the World Health Organization (Tang *et al*., 2004). In the current study, the incidence of periodontal disease in patients with newly diagnosed schizophrenia (length of illness: 1 year or less) in Taiwan was 65.7%, as assessed from dentist visit records. Although time, duration of schizophrenia, assessment tools, psychophysical condition and socioeconomic status were different in all three studies, the data crudely illustrated that in terms of duration of schizophrenia, the incidence of periodontal disease in patients with schizophrenia increases from 65.7% after approximately 1 year to 98.5% after 20 years from the schizophrenia diagnosis. The results possibly imply that early preventive measures could prevent the incidence of periodontal disease in approximately one-third of patients newly diagnosed with schizophrenia.

### Risk factors for periodontal disease in schizophrenia

Most evidence demonstrates that old age (Angelillo *et al*., 1995; Tang *et al*., 2004; Arnaiz *et al*., 2011; Gurbuz *et al*., 2011; Teng *et al*., 2011) and male sex (Gurbuz *et al*., 2011; Gopalakrishnapillai *et al*., 2012) are associated with significantly higher risks of periodontal disease in patients with schizophrenia. This evidence was obtained from patients with chronic schizophrenia and periodontal disease by using assessment tools such as the CPI. Therefore, we assessed the presence of periodontal disease by identifying dentist visits by patients within 1 year of a schizophrenia diagnosis. Differences between subjects and assessment tools may have led to the identification of young age and female sex as risk factors for treated periodontal disease in our study. In other words, young female patients in the early stage of schizophrenia may have an opportunity or good insights for treatment of periodontal disease with good prognosis. On the contrary, elderly male patients with chronic schizophrenia had poor periodontal health. The findings thus suggest that primary care staff should be more concerned about early prevention of periodontal disease in elderly male patients with schizophrenia because this population may not visit a dentist frequently and their periodontal disease could worsen when they enter the chronic stage of schizophrenia.

Low income level is a known crucial risk factor for periodontal disease in the general population (Pihlstrom *et al*., 2005). However, high income level was a risk factor for treated periodontal disease in patients with newly diagnosed schizophrenia – probably because the patients belonged to a high income level class in this study were well aware that dental visits are necessary for maintaining good oral hygiene. The financial burden of dental expenses was limited for the patients in our study because Taiwan's National Health Institutes provide most Taiwanese with basic dental care without large copayments (Teng *et al*., 2011). Notably, 2-year history of periodontal disease was the most vital risk factor for periodontal disease in the current study. Therefore, the incidence of periodontal disease increased dramatically from 9% at 2 years before schizophrenia diagnosis to 59.5% in the 1 year after. This increase might be ascribed to the relief that psychiatric treatment can provide to a patient – increasing in a patient's sense of reality and willingness to seek treatment for periodontal disease, leading to more than 6-fold dental visits – albeit with the adverse effects of antipsychotics.

### Antipsychotics and periodontal disease in schizophrenia

Little evidence is available about the adverse effects of antipsychotics and periodontal disease in patients with schizophrenia because most previous surveys had small-to-medium samples, and complex patterns of antipsychotics were prescribed to the study subjects (Hede, 1995; Gurbuz *et al*., 2011; Teng *et al*., 2011; Gopalakrishnapillai *et al*., 2012; Eltas *et al*., 2013). The current paper is among the few reports (Hede, 1995; Gopalakrishnapillai *et al*., 2012) that investigate the effects of antipsychotics on periodontal disease in patients with schizophrenia. It was impossible to assess the effect of each antipsychotic; we classified all antipsychotics and other medications into four categories: FGAs, SGAs, anticholinergics and antihypertensives. The findings of the current study illustrated that all four types of medication could accelerate development of periodontal disease, and the order of high to low risk is as follows: antihypertensives, FGAs, SGAs and anticholinergics. Extrapyramidal symptoms often occur with antipsychotics, particularly FGAs, so physicians prescribe co-medications such as anticholinergics or antihypertensives to alleviate these symptoms (Teng *et al*., 2011; Gopalakrishnapillai *et al*., 2012; Hu *et al*., 2016). Unfortunately, anticholinergics and antihypertensives can exacerbate the resultant periodontal disease. Therefore, clinicians should prescribe antipsychotics and other medications to the least extent possible under efficacious pharmacotherapy for schizophrenia.

Several reports have revealed that hyposalivation (xerostomia) may increase the risk of periodontal disease (Wagaiyu and Ashley, 1991; Hirotomi *et al*., 2006; Eltas *et al*., 2013; Mizutani *et al*., 2015); however, only one of these reports focused on patients with schizophrenia, the findings of which suggested that patients with schizophrenia who had reduced salivary flow rate (hyposalivation) due to antipsychotics were at higher risk of periodontal disease than those with increased salivary flow rate (hypersalivation) (Eltas *et al*., 2013) – a result similar to our study. We used the incidence of periodontal disease associated with antipsychotics and other medications as a surrogate because detailed data on the actual saliva flow rate of the patients with schizophrenia who were taking these medications were not available from the NHIRD. The benefit of using periodontal disease as a surrogate is switched to requiring medical attention. Our findings demonstrated that hyposalivation induced by FGA, anticholinergic and antihypertensive is potentially associated with increased risk of periodontal disease; hence, FGA-induced hypersalivation in periodontal disease is considered a protective factor. Although our statistical analyses could not provide sufficient information on the linked pharmacopathology between the adverse effects of saliva and periodontal disease, the data demonstrate how clinicians can reduce periodontal disease caused by the adverse effects of antipsychotics during pharmacotherapy for schizophrenia. The findings suggest that clinicians should avoid iatrogenic adverse effects on saliva while prescribing antipsychotics as far as possible and actively manage the adverse effects and ensure early dental referral. The possible underlying causal pharmacological mechanism must be determined in future studies.

### Strengths and limitations of the study

The major strength of the current study was the use of a large population-based cohort that enabled us to evaluate the relationship between antipsychotics and risk factors for periodontal disease in the early stages of schizophrenia. The findings may prove favourable to prevent the development of periodontal disease in patients with schizophrenia, especially the ethnic Chinese population. The well-determined temporal relationship between antipsychotic prescription and the occurrence of periodontal disease was another strength of this study. We obtained not only rigorous illness diagnoses but also correct medication information from the NHIRD. The study sample was identified based on the association between an ambulatory care expenditures database (ICD-9-CM codes and psychiatrists) and the prescription claims database (pharmacotherapy for schizophrenia) to increase the diagnostic accuracy of schizophrenia. We also considered significant covariates, including underlying diseases such as HIV/AIDS, diabetes mellitus, osteoporosis, chronic pulmonary diseases and a history of periodontal disease.

This study had several limitations. First, diagnoses of both periodontal disease and schizophrenia could have been underestimated, for instance, because of Berkson's bias, in which hospital cases and controls in a case–control study can be systematically different from one another because the combination of exposure to risk and disease occurrence increases the likelihood of admission. Hence, we surveyed treated patients with schizophrenia as well as the possible consequences of periodontal disease. Accordingly, treatment-naïve patients may have had only a limited effect on our analysis. Second, patient adherence to medications could not be evaluated because of the prescription claims database in this study. However, medication nonadherence would most likely have resulted in a nondifferentiated misclassification of exposure leading to possible underestimation of actual risk. Third, only treated periodontal disease indicated by the patients’ medical records was considered as the measure of periodontal disease occurrence in this study as opposed to all cases of periodontal disease wherein patients with schizophrenia developed periodontal disease but did not visit a dentist. Fourth, nonavailability of information on dietary, lifestyle and other potential risk factors for periodontal disease, such as illness severity, biochemistry data and patients’ unhealthy lifestyles such as tobacco consumption and poor oral hygiene (Albandar, 2002; Ramon *et al*., 2003; Scully and Bagan, 2004; Pihlstrom *et al*., 2005; Dumitrescu *et al*., 2008; Chu *et al*., 2012; Kossioni *et al*., 2012; Thomson *et al*., 2012; Genco and Borgnakke, 2013; Morales-Chávez *et al*., 2014; Hu *et al*., 2016) – was considered.

## Conclusions

In this paper, we highlight early prevention of periodontal disease in patients with schizophrenia. Based on our findings and previously reported evidence, we suggest that more care should be provided to men with schizophrenia who have a history of periodontal disease so as to prevent further periodontal degeneration. We also emphasise that in addition to paying more attention to development of periodontal disease, assessing oral health regularly, assisting with oral hygiene and lowering consumption of sugary drinks and tobacco, physicians should prescribe antipsychotics to the least extent possible and avoid iatrogenic adverse effects on saliva as far as possible under efficacious pharmacotherapy for schizophrenia.

## Availability of data and materials

In the current study, there are ethical or legal restrictions on sharing a de-identified data set. Therefore, we have provided contact information for a data access committee, see NHRID_SQL Generator http://sqlgen.net.nsysu.edu.tw/SQL_Generator/General_Searching.html (in English, cited on 2019/2/13).

## Appendix A

**Table A1.** ICD-9-CM codes and Anatomical Therapeutic Chemical [ATC] classification system codes used in this study

Main diseases	ICD-9-CM codes and ATC codes
Schizophrenia	295
Periodontal disease	523.0–523.5, 523.8, 523.9, 91001C, 91003C, 91004C, 91006C–91008C, 91009B, 91010B, 91011C–91013C, 91104C, 92033C, P4001C, P4002C
Exclusion comorbidity	ICD-9-CM codes
Osteoporosis	733.0
Diabetes mellitus	250
AIDS	042, 079.53, 795.71
Alcoholism	291, 303.0X, 303.9, 305.00–305.03, 357.5, 425.5, 535.30, 535.31, 571.0–571.5, 571.8, 571.9, 790.3, 980.0, 980.2, 980.8, 980.9, 977.3, V11.3
Chronic pulmonary disease	416.8, 416.9, 490, 491–495, 496, 500–505, 506.4, 508.1
Drug categories	ATC codes
FGAs	
Chlorpromazine (HCl)	N05AA01
Trifluoperazine (HCl) or (2HCl)	N05AB06
Thioridazine HCl	N05AC02
Haloperidol	N05AD01
Flupentixol (2HCl)	N05AF01
Chlorprothixene HCl	N05AF03
Thiothixene	N05AF04
Pimozide	N05AG02
Loxapine (succinate)	N05AH01
Sulpiride	N05AL01
SGAs	
Ziprasidone	N05AE04
Risperidone	N05AX08
Clozapine	N05AH02
Olanzapine (micronized)	N05AH03
Quetiapine (fumarate)	N05AH04
Amisulpride	N05AL05
Zotepine	N05AX11
Aripiprazole	N05AX12
Paliperidone	N05AX13
Anticholinergics	
Trihexyphenidyl HCl	N04AA01
Biperiden HCl	N04AA02
Diphenhydramine	R06AA52
Diphenhydramine HCl	R06AA52
Antihypertensives	
Furosemide	C03CA01
Propranolol HCl	C07AA05
Carteolol HCl	C07AA15
Atenolol	C07AB03
Enalapril maleate	C09AA02

**Table A2.** Hyposalivation and hypersalivation caused by antipsychotics and other medications

Drug categories	Adverse effects	
	Hyposalivation	Hypersalivation
FGAs		
Chlorpromazine (HCl)	+	0
Trifluoperazine (HCl) or (2HCl)	+	0
Thioridazine HCl	+	+
Haloperidol	+	+
Flupentixol (2HCl)	+	0
Chlorprothixene HCl	+	0
Thiothixene	+	0
Pimozide	+	0
Loxapine (succinate)	+	0
Sulpiride	+	0
SGAs		
Ziprasidone	+	0
Risperidone	+	+
Clozapine	+	+
Olanzapine (micronized)	+	+
Quetiapine (fumarate)	+	+
Amisulpride	+	0
Zotepine	+	0
Aripiprazole	+	0
Paliperidone	+	+
Anticholinergics		
Trihexyphenidyl HCl	+	0
Biperiden HCl	+	0
Diphenhydramine	+	0
Diphenhydramine HCl	+	0
Antihypertensives		
Furosemide	+	0
Propranolol HCl	+	0
Carteolol HCl	+	0
Atenolol	+	0
Enalapril maleate	+	0

+ = yes, 0 = no.
